# Nitrous oxide and the environmental case in anesthesia: scale, priority, and Brazilian evidence

**DOI:** 10.1016/j.bjane.2026.844845

**Published:** 2026-10-13

**Authors:** Ricardo Vieira Carlos, Britta Regli von Ungern-Sternberg, Vinícius Caldeira Quintão, Paulo Sergio Mateus Marcelino Serzedo, Marcelo Luis Abramides Torres

**Affiliations:** a Universidade de São Paulo (USP), Faculdade de Medicina (FM), Discipline of Anesthesiology, São Paulo, SP, Brazil; b Santa Casa de Misericórdia de Ribeirão Preto, Ribeirão Preto, SP, Brazil; c Child and Adolescent Health Service, Perth Children’s Hospital, Department of Anaesthesia and Pain Medicine, Nedlands, Western Australia, Australia; d The University of Western Australia, Institute for Paediatric Perioperative Excellence, Anaesthesia and Pain Management Program, Perth, Western Australia, Australia; e Perioperative Medicine Team, The Kids Research Institute Australia, Nedlands, Western Australia, Australia; f Division of Emergency Medicine, Anaesthesia and Pain Medicine, The University of Western Australia, Perth, Western Australia, Australia; g Universidade de São Paulo (USP), Faculdade de Medicina (FM), Department of Pediatrics, São Paulo, SP, Brazil; h Universidade de São Paulo (USP), Faculdade de Medicina (FM), Hospital das Clínicas (HC), Instituto da Criança e do Adolescente, São Paulo, SP, Brazil

Our responsibility as anesthesiologists does not end at the edge of the operating table. The gases we deliver leave the operating room, persist in the atmosphere for a century or more, and affect the health of populations we will never meet; the environment is therefore not an adjunct to our clinical duty but part of it. Seen in that light, the momentum to remove nitrous oxide (N_2_O) from operating rooms is a step in the right direction. It has placed the environmental footprint of anesthesia on the agenda of departments that had never previously considered it and has prompted concrete, measurable action where there had long been inertia. Yet the conversation has also become polarized, drifting towards a binary in which a clinician is expected either to defend N_2_O or to repudiate it. But in medicine, the right path is rarely found in black and white. A more useful question, surely, is not whether N_2_O should be used at all, but where its elimination fits among the many interventions available to a profession that genuinely wants to reduce its emissions. Our argument is one of proportionality: environmental effort, like any finite clinical resource, should be directed first to where it yields the greatest return, and a campaign organized around the most visible target is not the same as one organized around the largest.

It is worth stating clearly what this editorial does not address. There are legitimate, long-standing clinical concerns about N_2_O, including inactivation of vitamin B_12_ and methionine synthase with prolonged or repeated exposure, the hazards of chronic occupational exposure, and a contribution to postoperative nausea and vomiting. These questions have been examined in dedicated clinical investigations, including the large ENIGMA-II randomized trial, which found no significant increase in death or cardiovascular complications when N_2_O was added to general anesthesia in at-risk patients undergoing major non-cardiac surgery.^1^ The clinical risk-benefit calculus of N_2_O is a real and ongoing discussion that deserves its own forum. It is, however, separate from the environmental case, and conflating the two has muddied both: a clinical reservation about the drug is repeatedly invoked as evidence for an environmental policy, and an environmental anxiety is dressed up as a patient-safety imperative. Here we confine ourselves to the environmental argument, which rests on a single empirical question: where does clinical N_2_O actually sit in the global emissions ledger, and where would an equivalent quantum of effort yield greater environmental benefit?

The numbers are not ambiguous. Volatile (halogenated) anesthetics alone are estimated to account for approximately 0.1% of global warming, equivalent to roughly one million automobiles on the road.^2^ Worldwide, anesthetic N_2_O is estimated to contribute 1% to 3% of global N_2_O emissions.^2^ The healthcare sector as a whole is responsible for about 4.4% of global greenhouse gas emissions, and within an individual acute hospital, anesthetic gases account for roughly 5% of the institutional carbon footprint.^2^^,^^3^ These are not trivial figures, nor are they an argument for complacency. But they must be read against the dominant source of atmospheric N_2_O: the most recent global N_2_O budget attributes about 74% of the anthropogenic fraction of N_2_O emissions to agriculture, driven by synthetic nitrogen fertilizers and animal manure applied to soils.^4^ This is not a marginal contribution that clinical practice might meaningfully offset; it is the overwhelming human source. Against that backdrop, clinical N_2_O occupies a fraction of a fraction of the global N_2_O footprint (Fig. 1).

**Figure 1:**
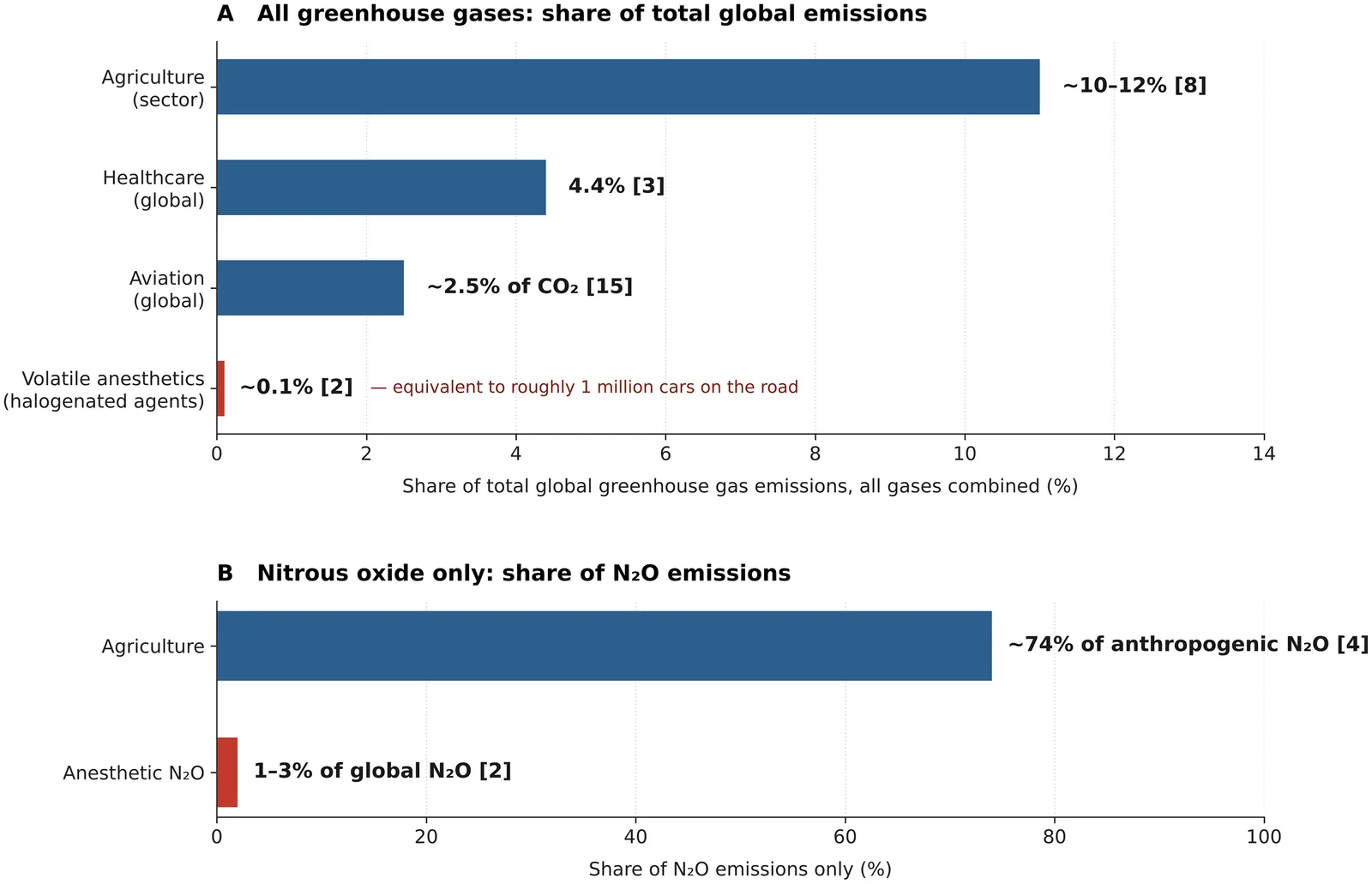
Anesthetic gases in the global emissions context. The two panels use different denominators. (A) Share of total global greenhouse gas emissions, all gases combined: agriculture accounts for ∼10–12%, reflecting its combined methane and N_2_O footprint;^8^ healthcare for about 4.4%;^3^ commercial aviation for approximately 2.5% of global CO_2_ emissions (more of total warming once non-CO_2_ effects are included);^15^ and volatile (halogenated) anesthetics for approximately 0.1% of global warming, equivalent to roughly one million cars on the road.^2^ (B) Share of N_2_O emissions only: agriculture accounts for ∼74% of anthropogenic N_2_O emissions,^4^ whereas anesthetic N_2_O contributes only 1–3% of total global N_2_O emissions.^2^ Clinical use therefore represents a fraction of a fraction of the global N_2_O burden. Numbers in brackets within the figure correspond to the reference list.

The primary leverage for atmospheric N_2_O lies in agriculture. Meta-analyses of agricultural soils show that emission factors vary substantially with the form, rate, and timing of nitrogen application, and field studies across the world's principal cereal crops confirm that inorganic fertilizer can substantially increase soil N_2_O emissions, leaving optimized nitrogen management as a large and largely unrealized mitigation opportunity.^5^^,^^6^ The arithmetic of the global budget therefore points unambiguously towards nitrogen-use efficiency in food production as the intervention with the greatest planetary return. This does not absolve anesthesiologists of responsibility for their own emissions, but it sets the scale against which clinical N_2_O must be judged.

The chemistry underscores the point. N_2_O has a 100-year global warming potential of approximately 273 times that of carbon dioxide and an atmospheric lifetime of roughly 109 years. It is now the single most important ozone-depleting substance still emitted at scale.^7^^,^^8^ None of this is a reason to treat clinical use as the appropriate target. A gas can be both individually potent and collectively minor as a clinical emission, and N_2_O is exactly that: its environmental significance is the product of mass emitted and warming potential, and the mass emitted from operating rooms is small relative to the rest of healthcare and, overwhelmingly, to the rest of the economy.

The most important feature of N_2_O's hospital footprint, however, is that it does not arise primarily from clinical use. Across multiple international health systems, between 47.2% and 99.8% of the N_2_O a hospital procures is lost to the atmosphere through leaks in central pipeline (manifold) systems, without ever reaching a patient.^9^ A United Kingdom multicenter study quantified this gap directly: across the participating hospitals, clinical use accounted for only about 18% of the N_2_O procured, implying that more than four-fifths was lost before use; comparable work from Australia estimated roughly 77% pre-utilization loss.^7^^,^^10^ The waste is structural – embedded in reticulated infrastructure that leaks continuously at joints and fittings and compounded by the venting of residual gas when partially full cylinders are exchanged – and it has nothing to do with how judiciously any individual anesthesiologist prescribes the gas. This relocates the problem from the bedside to the building, and from clinical behavior to procurement and engineering: a department could reduce its clinical N_2_O use to zero and still leak the great majority of what the hospital buys.

The implication is that the greatest environmental gains come not from restricting clinical use but from decommissioning central pipelines and replacing them with point-of-care portable cylinders. This is now the explicit recommendation of the World Federation of Societies of Anaesthesiologists, whose 2026 statement frames the change as a shift from high-volume waste to low-volume, precision point-of-care use rather than as elimination from clinical care; the American Society of Anesthesiologists has issued a parallel statement. Crucially, evidence that this works has been generated in Brazil: a quality improvement initiative across two quaternary hospitals in São Paulo achieved an 82.5% reduction in anesthesia-related N_2_O CO_2_e emissions through an educational program combined with workstation-level disconnection of the central pipeline, with N_2_O preserved selectively for pediatric inhalational induction and no adverse patient outcomes attributed to the change in delivery.^11^ Notably, residual post-intervention emissions in that study, at about 23 kg CO_2_e per case, remained roughly threefold higher than benchmarks from settings without pipeline-related leakage – a finding that reinforces rather than weakens the central conclusion: the supply pipeline is the dominant source, and even an aggressive workstation-level intervention leaves residual upstream losses that full decommissioning can capture. The one element of that intervention we would now qualify is the retention of N_2_O for inhalational induction, for the reasons set out below.

This argument extends well beyond the operating room, which matters for any policy that aspires to be comprehensive. Clinical N_2_O is used not only in surgical anesthesia but also in obstetric analgesia, pediatric and dental sedation, and prehospital and emergency settings, all of which rely on the same upstream supply infrastructure. Reducing pipeline-related losses therefore captures benefits across the entire spectrum of medical N_2_O use, and it does so without removing a tool that retains genuine value in specific contexts: brief pediatric interventions such as intravenous cannulation, dressing changes, and minor wound care, and labor analgesia, where the alternative is often physical restraint or a general anesthetic and where the volumes involved are small. A pipeline-focused strategy is, in this sense, both more effective and less disruptive than prohibition: it eliminates waste while preserving care.

One pediatric indication, however, should be conceded rather than defended. N_2_O does not facilitate tranquil mask placement in an unwilling child and does not meaningfully accelerate inhalational induction. Its presence in the fresh gas precludes adequate preoxygenation – a narrow margin to surrender in patients whose smaller functional residual capacity, higher oxygen consumption, and greater risk of laryngospasm already shorten the time to desaturation.^12^ Practice has moved accordingly: a quality improvement program spanning 33,285 inhalational inductions reduced N_2_O use from 80% to below 20% and cut maximum fresh gas flows by 28%, with induction times and behavior unchanged.^13^ That is what one would expect if N_2_O's contribution to mask induction were largely a matter of tradition rather than pharmacology. Abandoning it in this setting costs nothing clinically and should be uncontroversial – and it was achieved through education and changed defaults rather than prohibition, which is precisely the point.

The case for prioritizing infrastructure over prohibition is strengthened by what we know about how Brazilian anesthesiologists already use the gas. A recent nationwide cross-sectional survey conducted within a large private hospital network and published in this journal captured responses from 941 of 1,238 eligible anesthesiologists (a 76% response rate).^14^ Clinical N_2_O use in adults is already low and declining: 59.3% reported never using it in adult patients, and only 14.5% reported frequent or constant use.^14^ Pediatric practice differed – 38.9% reported frequent or constant use in children, compared with 27.5% who never used it – reflecting the historical role of N_2_O in inhalational induction, a practice that the pediatric evidence no longer supports.^14^ Eighty percent reported that their use had fallen over their careers, indicating a substantial behavioral shift already underway without any categorical ban, while 34.5% were unaware of N_2_O's contribution to climate change.^14^

Two implications follow. First, because the clinical trend is already moving in the right direction, the marginal return on a formal ban on adult use would be modest. Second, the persistence of pediatric use calls for a more discriminating response than a ban. When that use is inhalational induction, it should be discontinued on clinical as well as environmental grounds, and the international trend is already sharply downward.^12^^,^^13^ When it is brief procedural analgesia – cannulation, dressing changes, minor wound care – or labor analgesia, it reflects genuine clinical value: precisely the low-volume, high-justification use that a portable-cylinder model is designed to preserve. A policy that decommissions the pipeline while retaining cylinder-based N_2_O for those indications aligns environmental and clinical interests rather than setting them against one another and is far more likely to be accepted by the clinicians who must implement it.

If clinical N_2_O emissions are small, where should comparable effort go? Comparisons with other sources are useful for prioritization, even if they are not, in themselves, a license for inaction. A single intercontinental flight emits on the order of one to three tonnes of CO_2_e per passenger, and commercial aviation as a whole contributes roughly 2.5% of global CO_2_ emissions and more of total warming once non-CO_2_ effects are included.^15^ The point is not that anesthetic emissions are acceptable because others are larger – that would be the very error of false exemption we are warning against – but that a profession serious about its footprint should sequence its actions by tractable magnitude rather than by symbolic visibility. That sequencing points inward as well as outward. Within our own operating rooms, single-use plastics, over-specified procedure packs, unrecycled packaging and the waste stream generated by every case are substantial, largely unmeasured and entirely within our control, and reducing them requires no trade-off against clinical care at all.^16^

On that logic, several interventions deliver substantially more benefit than withdrawing N_2_O from the adult formulary. The first is decommissioning central N_2_O pipelines and replacing them with closed portable cylinders, which addresses the dominant source of loss. The second is eliminating desflurane, whose global warming potential of approximately 2,540 is roughly an order of magnitude greater than that of N_2_O;^8^^,^^17^ many departments removed it from the formulary years ago with no clinical detriment, and doing so eliminates a large per-case emission at almost no cost, given the availability of sevoflurane and total intravenous anesthesia. The third is optimizing fresh gas flow and expanding low-flow and intravenous techniques, since volatile-agent consumption scales directly with fresh gas flow, halving the flow halves the emission, and reductions of one-half to three-quarters are achievable without changing agent.^17^ The fourth is routine integrity checks of portable cylinder valves and connections, paired with institutional clinician-education programs that the Brazilian quality-improvement literature has shown to be effective.^11^ None of these requires removing a clinically useful agent from the formulary.

The desflurane phase-out is instructive because it illustrates what proportionate regulation looks like. Scotland removed desflurane from routine use in 2023, the first health system to do so; NHS England completed its decommissioning in 2024; and the European Union's revised regulation on fluorinated greenhouse gases prohibits routine desflurane use from January 2026, with an explicit exemption when no other agent is medically suitable.^18^ Regulators acted because the magnitude of a single agent's emissions justified it: desflurane's per-case footprint is large, its clinical advantages are narrow, and substitutes are readily available – the conditions under which prohibition is the proportionate instrument. The modest clinical contribution of N_2_O does not approach that threshold, and the structure of its waste – overwhelmingly pre-utilization pipeline loss rather than clinical administration - points to a different remedy. To treat N_2_O as though it were desflurane is to misapply a sound precedent.

A thoughtful case can still be made for elimination on grounds other than tonnage: that a clean break is administratively simpler than maintaining a cylinder supply for a few indications; that the precautionary principle favors removing a potent, ozone-depleting gas wherever feasible; and that the symbolic value of the specialty renouncing N_2_O might catalyze wider change. These are not trivial points. But simplicity that removes a valuable option for children and laboring women is a false economy; precaution is better served by eliminating the large fraction of procured gas now vented unused rather than the small fraction delivered to patients; and symbolism that diverts attention from larger sources is, in the end, a cost. The most credible signal the specialty can send is not the renunciation of a single agent but a demonstrated willingness to follow the evidence wherever the emissions actually are.

The equity dimension reinforces the same conclusion. In resource-constrained systems, including much of the public sector in low- and middle-income countries, managerial attention, capital, and clinical goodwill for environmental work are scarce, and the opportunity cost of misdirected effort is correspondingly high. Brazil, with one of the world's largest public health systems and a sophisticated private sector, is well positioned to model a proportionate approach that delivers large, verifiable reductions without rationing care and to export it to other middle-income systems facing the same constraints.

The next step for the anesthesia community is to align local interventions with evidence on where emissions originate. For N_2_O, that evidence is unusually clear and actionable: the dominant hospital source is a leaking supply infrastructure; the dominant global source is agriculture; and clinical administration – already declining in adult practice and clinically valuable in selected pediatric and obstetric use – is a small contributor by every available measure. Environmental leadership in our specialty will not come from the categorical removal of an agent that retains specific clinical value, but from priority-ranked action that addresses the largest and most tractable sources first: decommissioning pipelines, eliminating desflurane, lowering fresh gas flows, and educating clinicians. Proportionality is not an excuse for inaction; it is the discipline that makes our action count.

## Data availability statement

No new data were generated or analyzed for this Editorial. All data discussed are derived from the cited publications.

## Funding

This editorial received no specific grant from any funding agency in the public, commercial, or not-for-profit sectors.

## Declaration of generative AI and AI-assisted technologies in the manuscript preparation process

During the preparation of this work, the author(s) used Claude (Anthropic) to generate Figure 1. The author(s) reviewed and edited the output as needed and take full responsibility for the content of the published article.

## Declaration of competing interest

BSvUS is part-funded by the Stan Perron Charitable Foundation and through a National Health and Medical Research Council Investigator Grant (2009322). VCQ is supported by a Research Productivity Fellowship (Bolsa de Produtividade em Pesquisa, Level C) from the Conselho Nacional de Desenvolvimento Científico e Tecnológico (Brazilian National Council for Scientific and Technological Development, CNPq). VCQ and RVC are associate editors of the Brazilian Journal of Anesthesiology.
